# Rationalizing the Decavanadate(V) and Oxidovanadium(IV) Binding to G-Actin and the Competition with Decaniobate(V) and ATP

**DOI:** 10.1021/acs.inorgchem.0c02971

**Published:** 2020-11-30

**Authors:** Giuseppe Sciortino, Manuel Aureliano, Eugenio Garribba

**Affiliations:** † Dipartimento di Chimica e Farmacia, Università di Sassari, Via Vienna 2, I-07100 Sassari, Italy; ‡ Institute of Chemical Research of Catalonia (ICIQ), Avgda. Països Catalans, 16, 43007 Tarragona, Spain; § CCMar, FCT, Faculdade de Ciências e Tecnologia, Universidade do Algarve, 8000-139 Faro, Portugal

## Abstract

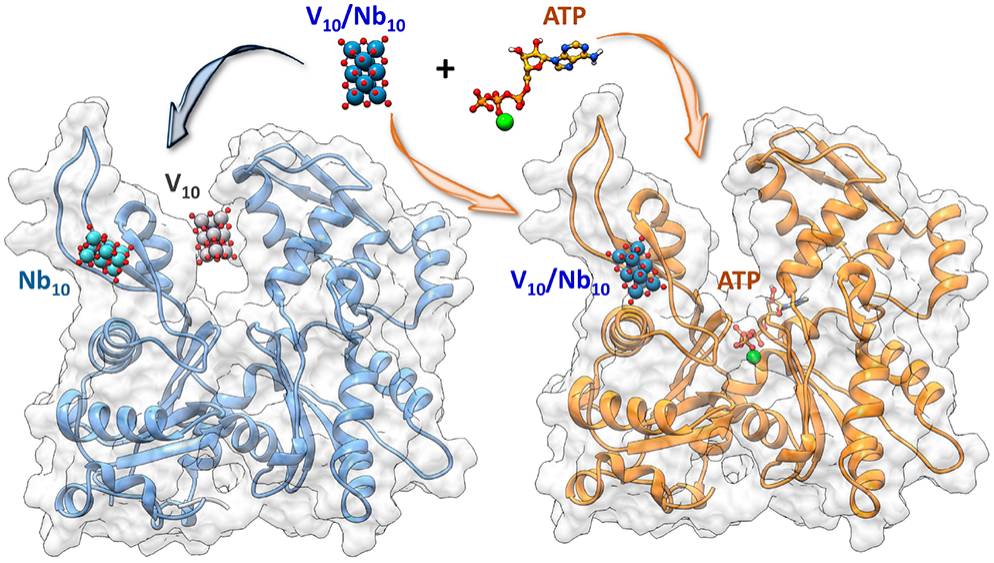

The experimental data collected over the past 15 years on the interaction of decavanadate(V) (V_10_O_28_^6–^; V_10_), a polyoxometalate (POM) with promising anticancer and antibacterial action, with G-actin, were rationalized by using several computational approaches (docking, density functional theory (DFT), and molecular dynamics (MD)). Moreover, a comparison with the isostructural and more stable decaniobate(V) (Nb_10_O_28_^6–^; Nb_10_) was carried out. Four binding sites were identified, named α, β, γ, and δ, the site α being the catalytic nucleotide site located in the cleft of the enzyme at the interface of the subdomains II and IV. It was observed that the site α is preferred by V_10_, whereas Nb_10_ is more stable at the site β; this indicates that, differently from other proteins, G-actin could contemporaneously bind the two POMs, whose action would be synergistic. Both decavanadate and decaniobate induce conformational rearrangements in G-actin, larger for V_10_ than Nb_10_. Moreover, the binding mode of oxidovanadium(IV) ion, V^IV^O^2+^, formed upon the reduction of decavanadate(V) by the –SH groups of accessible cysteine residues, is also found in the catalytic site α with (His161, Asp154) coordination; this adduct overlaps significantly with the region where ATP is bound, accounting for the competition between V_10_ and its reduction product V^IV^O^2+^ with ATP, as previously observed by EPR spectroscopy. Finally, the competition with ATP was rationalized: since decavanadate prefers the nucleotide site α, Ca^2+^-ATP displaces V_10_ from this site, while the competition is less important for Nb_10_ because this POM shows a higher affinity for β than for site α. A relevant consequence of this paper is that other metallodrug–protein systems, in the absence or presence of eventual inhibitors and/or competition with molecules of the organism, could be studied with the same approach, suggesting important elements for an explanation of the biological data and a rational drug design.

## Abstract 2

Four putative binding sites in globular G-actin (named α, β, γ, and δ) were identified for two biologically active polyoxometalates (POMs): decavanadate(V) (V_10_) and decaniobate(V) (Nb_10_). Among the binding sites, V_10_ prefers the catalytic nucleotide site (site α), while Nb_10_ prefers the site β. ATP prevents mostly the binding of V_10_ than Nb_10_, competing with decavanadate for the site α.

## Introduction

After the discovery of cisplatin, inorganic medicinal chemistry is a field of extensive research. Potential antitumor drugs based on Pt, Ru, and Cu, antimicrobial and antiparasitic agents based on Sb, Ag, As, and Fe, antiarthritis on Au, antiulcers on Bi, and antivirals based on Co and Hg were proposed. In addition, diagnostic metallodrugs of Gd, Tc, and In were also developed.^1,2^ Similarly, vanadium compounds have long been studied as potential antidiabetic drugs and, more recently, as anticancer, antiparasitic, antiviral, antiHIV, and antituberculosis agents.^3^

Interaction with proteins plays a crucial role in the transport and activity of metallodrugs in the organism, both for their high affinity toward the metals or high concentration in the bloodstream and cellular environment. Unfortunately, in most of cases, the experimental data cannot be rationalized through a description at the molecular level of this interaction.^4^ Instrumental methods often give only information about the biological effects of a metallodrug after the binding with a target protein but present severe limitations to unveil the type of interaction and the binding modes and sites or to ascertain the competition between the metallospecies and other biomolecules of the organism.

Decavanadate (V_10_O_28_^6–^; V_10_) and decaniobate (Nb_10_O_28_^6–^; Nb_10_) are members of a large family of polyanionic clusters formed by group V and VI metals that are known as polyoxometalates (POMs). POMs have been found to have a wide range of biological activities such as antidiabetic, antibacterial, antiprotozoal, antiviral, and anticancer, which have sparked interest in their use as bioinorganic drugs.^5^ Recently, it has been demonstrated that POMs function as indirect activators of a G protein-coupled receptor.^6^ Moreover, POMs have also seen applications in other fields of research such as aerobic oxidation of starch, smart glasses, solar-driven photocatalysis, protein crystallography, and synthesis of metal–organic frameworks (MOFs);^7^ for example, Nb_10_ can be used as building block to prepare 1D, 2D, and 3D frameworks.^8^

Based on an analysis of the antibacterial activity and POM structures, putative mode of actions toward potential targets were suggested and a structure–activity relationship was determined for a series of POMs against *Helicobacter pylori* and *Streptococcus pneumoniae*.^5e^ The activity of POMs are found to be bacteria-dependent: for *Streptococcus pneumoniae* the most active POMs were polyoxovanadates (POVs), especially V_10_, while for *Helicobacter pylori* most of POMs are effective.^5e^

The group of Aureliano extensively studied the influence of POMs, especially POVs and polyoxotungstates (POTs), on diverse biochemical mechanisms.^9^ In many cases, these POM-associated biological activities are the result of POM–protein/enzyme interactions, which are mainly of electrostatic nature as evidenced by crystallographic studies showing that the negatively charged metal clusters are generally found within or at positively charged regions of proteins.^10^ However, also covalent interactions between biomacromolecules and POMs are possible.^10,11^ In this scenario, the inhibition of certain enzymes by POMs can trigger the impairment of vital cell functions.^10,11^

Among the possible cellular targets for POMs, actins are worth being considered. Actins are found in all life kingdoms and are responsible for many biologically essential processes, such as muscle contraction, cell adhesion, division, and migration, thus assigning them important roles in health and diseases.^12^ Globular actin (G-actin; 42 kDa), the actin monomer, is one of the most abundant proteins in cells, being involved in numerous cellular processes. Previously, it was shown that V_10_–G-actin interaction affects the protein structure and dynamics and also influences its transformation to filamentous actin (F-actin, the polymerized form of actin).^11^ Combining our experience regarding V_10_–G-actin system^11,13−15^ and our expertizes in computational characterization of metallospecies–protein binding,^16^ we further pursue the interaction between decavanadate and G-actin to deduce potential targets and provide possible modes of the biological activity of POMs.

Despite V_10_ and Nb_10_ being isoelectronic and isostructural isopolyoxoanions, V_10_ is redox-active and labile in aqueous solution, while Nb_10_ is kinetically inert and redox stable.^17^ Although there has been growing interest in niobium chemistry in the past decade, research on decaniobate and its interplay with biomolecules and/or biological effects is still scarce.^9h,18,19^ In the systems with Ca^2+^-ATPase, Nb_10_ prevents the binding of V_10_, suggesting the same target(s) in the protein structure;^18^ moreover, both of them cause comparable conformational changes.^9h,19^ In addition, Nb_10_ constitutes an excellent model to explore the interplay between its V_10_ analogue and biologically vital proteins such as actin.

Even though some studies were performed on the systems containing POMs and actin, up to now the detailed knowledge of the molecular basis of V_10_–G-actin interactions and the possible involvement in protein polymerization is lacking. In fact, the exact mechanism of action of POMs is still elusive and thus also the POM’s target enzymes, which are responsible for the observed biological effects. Molecular modeling could represent a valid tool to fill this gap and help to understand cellular processes providing valuable clues for the rational design of new metal-substituted POMs with specific interaction properties, pushing forward the comprehension of the molecular events governing POMs–protein binding. Even though the computational techniques have been largely applied to study the chemistry of POMs,^20^ only few examples of molecular modeling application to POM–protein interaction have been published so far.^21^

On the basis of these premises, in this paper we tried to rationalize—using several computational approaches—the experimental data on the V_10_–G-actin systems, collected over the past years in the literature, and compare the findings with the isostructural Nb_10_. In particular, the aims of this study are (i) to attain an understanding, at the molecular level, of the interaction of V_10_ and Nb_10_ with G-actin starting from the available results already obtained by several spectroscopic techniques;^11,13−15^ (ii) to fully characterize the binding sites of G-actin for V_10_ and Nb_10_, determine the number and type of neighbor atoms around POMs, and evaluate their stabilization through secondary interactions; (iii) to compare the behavior of V_10_ and Nb_10_; (iv) to predict the binding site(s) of the reduction product of V_10_, the oxidovanadium(IV) ion, V^IV^O^2+^; and (v) to study the V_10_/Nb_10_–G-actin system in the presence of ATP to obtain a more accurate description of the mechanisms involved and possible competition/inhibition effects.

## Computational Section

### DFT Calculations

V_10_O_28_^6–^ and Nb_10_O_28_^6–^ geometry and harmonic frequencies were computed through Gaussian 09 (revision D.01)^22^ by using the B3LYP functional including Grimme’s empirical correction for dispersion.^23^ V and Nb atoms were described with the scalar-relativistic Stuttgart–Dresden SDD double-ζ basis set complemented with f polarization functions and its associated pseudopotential.^24^ The 6-31g(d,p) basis set was used for the oxygen atoms. The structures of POMs were optimized in water described by the SMD continuum model.^25^ Point charges of the complete POMs were derived with the RESP^26^ (Restrained ElectroStatic Potential) model. The DFT level of theory and RESP model to assign the atomic charges were selected following the protocol proposed to derive AMBER and TIP3P force fields for molecular dynamics (MD).^27^

The refinement of the oxidovanadium(IV) adducts at the nucleotide site (site α), found by dockings, was performed by cutting out the region with the V^IV^O^2+^ ion and neighboring interacting amino acid side chains and freezing the backbone atoms as reported by Siegbahn and Himo.^28^ The geometry relaxation and Δ*E* calculations were performed by using the doublet state in unrestricted simulations at the B3P86/6-311++g(d, p) level of theory and describing water within the framework of the SMD model^25^ which gave excellent results for other V^IV^–protein systems.^16f,16g,29^ For the geometry optimization of V^IV^ complexes, it was shown that B3P86 works better than other functionals like B3LYP.^30^ Frequency calculations were computed to ensure that the structures were a minimum in the potential energy surface.

The prediction of the ^51^V hyperfine coupling tensor **A** was performed on the optimized structures with Gaussian 09^22^ by using BHandHLYP functional and 6-311+g(d) basis set.^31^ The spin contamination was negligible, and the eigenvalue of the spin operator ⟨*S*^2^⟩ was 0.76 for all the calculations, very close to the exact value of 0.75. This is in agreement with the results obtained for hybrid functionals.^32^ The theory background can be found in refs (32b and 33).

### Molecular Dynamics

The apo form of monomeric actin, G-actin, was built by removing Ca^2+^-ATP ligand and adding non-characterized regions 39–51 and 1–5 with Modeler^34^ on the unique native X-ray structure of mononuclear G-actin (PDB: 3hbt(35)). In the case of ATP bound simulations and competition studies, the Ca^2+^-ATP present in the X-ray structure has been retained. Molecular dynamics simulations were set up with Xleap,^36^ which was instructed to solvate the protein with a cubic box of pre-equilibrated TIP3P water molecules and balance the total charge with Na^+^ ions (ions94.lib library). The AMBER14SB force field^37^ was used for the protein, while V- and Nb-bonding force constants and equilibrium parameters were obtained through the Seminario method with the MCPB.py.^38^ Ca^2+^-ATP parameters from Meagher et al.^39^ and Bradbrook et al.^40^ have been used.

During MDs, the solvent and the whole system were sequentially submitted to 3000 energy minimization steps. Then, thermalization of water molecules and side chains was achieved by increasing the temperature from 100 up to 300 K. MDs under periodic boundary conditions were performed during 200 ns with OpenMM engine^41^ through OMMProtocol.^42^

Analysis of the trajectories was performed by means of cpptraj implemented in ambertools16.^36^ The MD trajectory was considered converged when a stable exploration of the conformational space was achieved; in particular, a stable conformation or a pool of relative stable conformations visited for a statistically consistent number of times were considered as convergence indicators.^43^ Considering the α carbons of the G-actin backbone, the root-mean-square deviation (RMSD) from the minimized structure, all-to-all frames RMSD,^44^ and cluster counting^45^ analysis were performed. Moreover, to ensure that dynamic transitions occur between different conformations, a principal component analysis (PCA) was performed plotting the two principal modes relative to each other.^44^ The quality threshold (QT) clustering algorithm was used to generate representative clusters taking the RMSD between frames as distance metric.^46^ The representative structure of the most populated cluster was used in the further structural analysis.

### Docking Calculations

Docking calculations to the G-actin were performed through GOLD 5.8 software^47^ on the most sampled structure coming from MD trajectories. G-actin was docked with V_10_ and Nb_10_ optimized structures considering the whole rigid protein.

Dockings of V^IV^O^2+^ moiety were performed through GOLD 5.8^47^ on the regions selected in the preliminary analysis by BioMetAll,^48^ instructed to find latent binding motifs with a minimum of two coordinating residues considering His, Asp, and Glu as potential donors. G-actin was docked with V^IV^O^2+^, V^IV^O(H_2_O)^2+^, and V^IV^O(H_2_O)_2_^2+^ moieties, obtained from the optimization of the oxidovanadium(IV) aquaion, [V^IV^O(H_2_O)_4_]^2+^. The equatorial positions were activated by replacing the equatorial water(s) with dummy hydrogen atoms according to what was recently established.^16a,16c,16e,16h^ All dockings were computed considering both the protonation states at δ and ε nitrogens of the His imidazole ring. Genetic algorithm (GA) parameters have been set to perform a minimum of 100000 operations during 50 GA runs for covalent dockings and 100 GA runs for non-covalent dockings. The other parameters of GA were set to default. The scoring (*Fitness* of GoldScore, *F*) was evaluated by applying the modified versions of GoldScore scoring function, which was validated in previous published papers.^16a,16c,16e^ The best solutions (binding poses) were evaluated through three main criteria: (i) the mean (*F*_mean_) and the highest value (*F*_max_) of the scoring associated with each pose, (ii) the population of the cluster containing the best pose, and (iii) the position in the *Fitness* ranking of the computed cluster.

The POMs docking solutions were refined sending for each best proposal a 200 ns of MD and Δ*G*_bind_ quantified by molecular mechanics-generalized Born surface area (MM-GBSA) including quasi-harmonic correction for entropy.^49^ Average values have been obtained from 100 homogeneously distributed frames of the whole MD trajectories. The following parameters were used: igb = 5 and saltcon = 0.100.

## Results and Discussion

### Determination of the Binding Sites

Experimental data reported so far pointed out that decavanadate anion (V_10_) interacts strongly with G-actin.^11,13,14,50^ However, up to now, even if the biological effects are clear, the binding site(s) were not determined, and this datum is lacking in the literature. Nevertheless, this information could be important and related to the subsequent reduction process to the oxidovanadium(IV) cation, V^IV^O^2+^,^13^ and could be very useful to gain insight for the rational design of new metal-substituted POMs (i.e., with two or more metals in their structure) with specific interaction with proteins.

**Figure 1:**
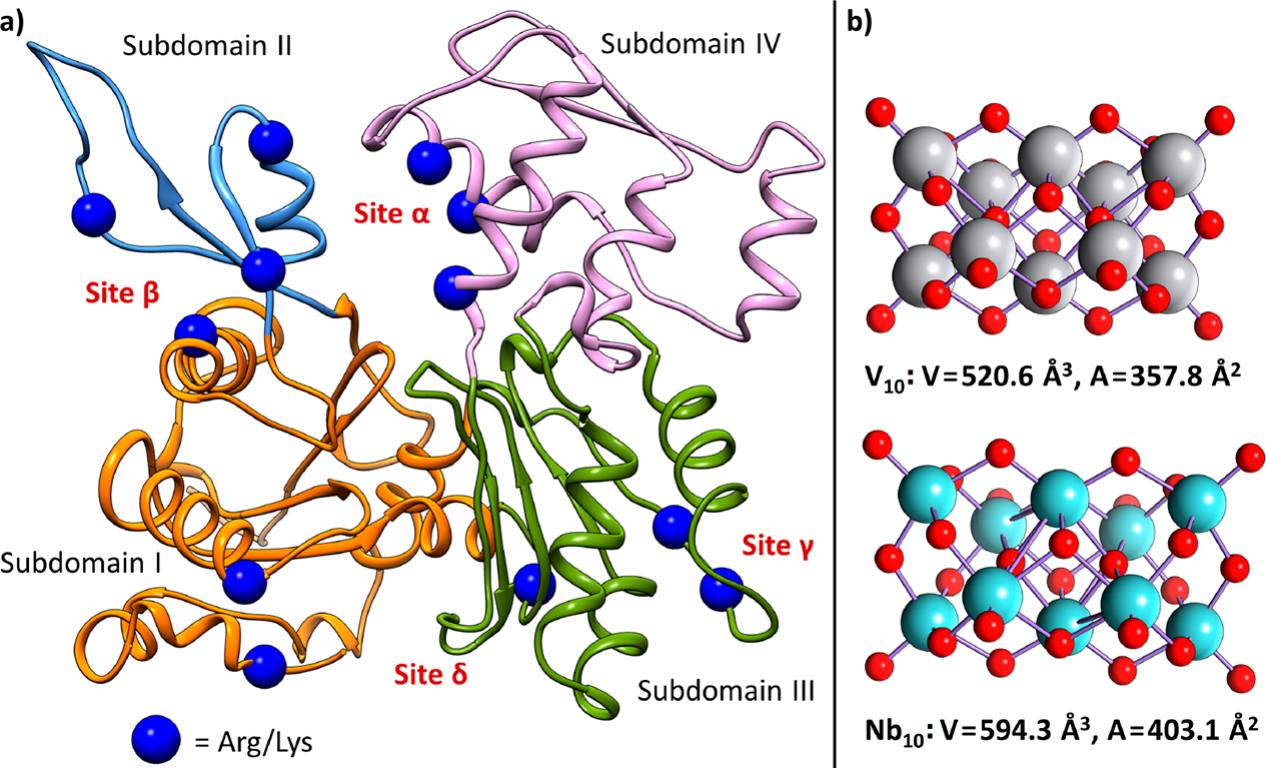
(a) The four binding sites found by docking for V_10_ and Nb_10_ in the structure of G-actin. The positively charged side chains of Arg and Lys, which interact with POMs in each site, are depicted as blue balls. Subdomains (I to IV) of G-actin are shown with different colors (orange, blue, purple, and green). (b) Comparison of the DFT optimized structure of V_10_ and Nb_10_. Volume (*V* in Å^3^) and surface area (*A* in Å^2^) were computed by considering van der Waals (vdW) radii. V is shown in gray, Nb in mint, and O in red.

To throw light on the unknown aspects, in the first step a blind docking screening was performed on the equilibrated apo structure of G-actin, searching for putative binding sites for V_10_ and Nb_10_. The results of this rigid docking essay suggest the presence of four potential binding sites, each of them involving at least two positive residues of Lys or Arg: the catalytic nucleotide site (here named site α), where the hydrolysis of ATP occurs,^51^ located in the cleft of the enzyme at the interface of subdomains II and IV; the site β at the interface of subdomains I and II; the site γ at the surface of subdomain III; and the site δ at the C-terminus in the small cleft between subdomains I and III (Figure 1a). After this preliminary stage, further docking calculations on the identified regions were performed by considering the flexibility of the side chains: similar results for both POMs were obtained with scoring (*F*_max_) ranging from 24.8 to 36.4 Gold Score *Fitness* units. For the site α, the pose (solution) with the best scoring is stabilized by hydrogen bonds (H-bonds) with Arg207, Arg211, Arg62, Thr204, Gln59, and Tyr69 residues. The site β, with similar scoring, presents H-bond with Lys68, Arg37, Lys50, and Lys84. The sites γ and δ show lower scorings and H-bonds with Arg147, Lys329, Thr148, and Asn297 or Arg113, Lys374, and Tyr170, respectively (Figure 1a). Comparison of the DFT optimized structure of V_10_ and Nb_10_ is shown in Figure 1b; Nb_10_ displays a slightly wider volume and surface area than its vanadium homologue V_10_ and, moreover, accumulates more surface negative charge (Figure S1 of the Supporting Information). However, since only slight differences in the behavior of V_10_ and Nb_10_ were observed, because of the similarity between the two structures, dockings can be useful at most for defining the possible binding regions but do not allow for ascertaining their relative affinities for the four sites. To move beyond docking and obtain reliable values for Δ*G*_bind_ for the two POMs, MDs were performed and residence time analysis and MM-GBSA calculations carried out.

### Conformational Changes Induced by V_10_ and Nb_10_ and Relative Stability of the Binding Sites

**Figure 2:**
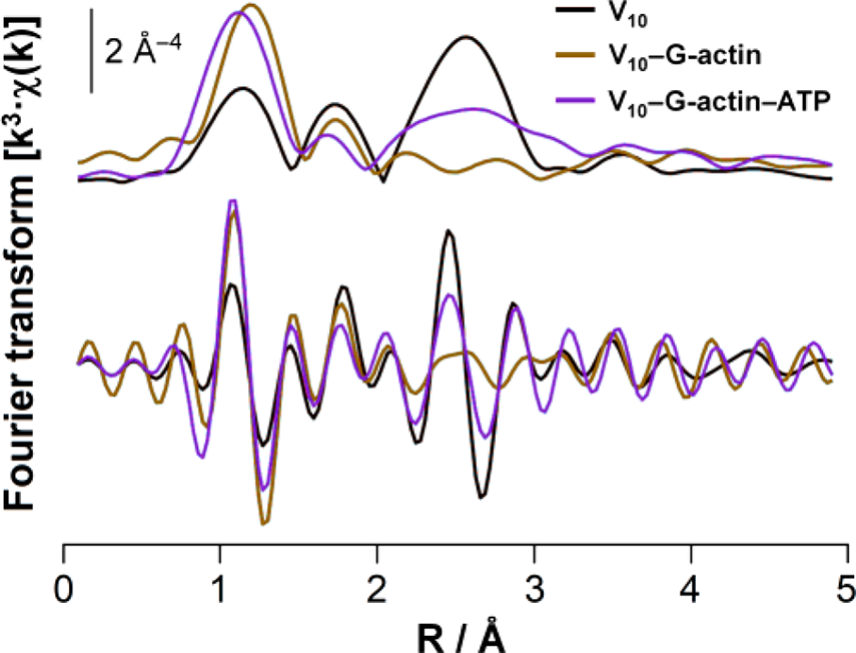
EXAFS profile of the systems containing V_10_ (in black), V_10_–G-actine (in brown), and V_10_–G-actin–ATP (in purple). Adapted from ref (15).

It was previously described in 2009 that the experimental exchange rate and apparent dissociation constant for ATP measured from fluorescence decrease (Figure S2) indicated that V_10_ binding induced an opening of the G-actin cleft.^13^ Subsequently, in 2011, the ^1^H NMR spectra of G-actin treated with V_10_ suggested that the latter causes major alterations in the protein structure (Figure S3);^50,52^ in contrast, no significant changes in the ^51^V NMR signals were observed for other vanadates, such as monomeric (H_2_V^V^O_4_^–^/HV^V^O_4_^2–^; V_1_) and tetrameric species (V_4_O_12_^4–^; V_4_).^13^ More recently, EXAFS and XANES experiments established that the interaction  V_10_–G-actin triggers a protein conformational reorientation that causes an oxidation of Cys residues and formation of the V^IV^O^2+^ cation (Figure 2).^15^

The change of actin conformation was further confirmed by circular dichroism (CD) spectroscopy.^53^ Upon addition of V_10_ to G-actin, the melting points determined from the CD melting curves were observed at lower values compared with the system with ATP at Tm_1_ 31.7 vs 48.9 °C and at Tm_2_ 51.7 vs 64.3 °C. The decrease (in the range 12.6–17.2 °C) in the protein melting temperature could probably be due to actin conformational changes and/or partial protein unfolding induced by V_10_.^53^

**Figure 3:**
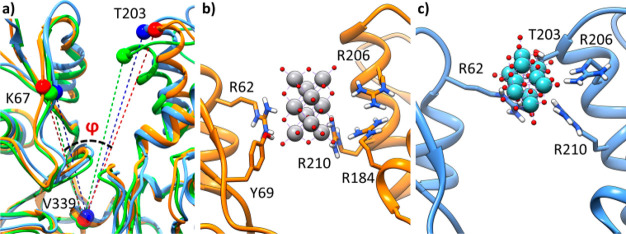
Molecular dynamics (MD) most sampled structures showing (a) the cleft opening upon the POMs binding (apo G-actin, V_10_–G-actin, and Nb_10_–G-actin are shown in green, orange, and blue, respectively), (b) binding mode of V_10_ at the site α, and (c) binding mode of Nb_10_ at the site α. The interacting side chains are explicitly depicted.

To assess the effect of the two POMs on the protein folding, subsequent MD simulations were performed starting from V_10_ and Nb_10_ bound to each of the four possible sites and compared with the unbound state of G-actin. Concerning the site α, adducts are highly stable both for V_10_ and Nb_10_ during the whole explored trajectory (200 ns), and the folding of the protein is generally retained. It must be noted that a slight opening of the nucleotide binding site is observed after the interaction with V_10_ and Nb_10_. Subdomain IV moves from subdomain II to accommodate the POMs. This movement can be described with the angle φ, defined as C(α)_Lys67_–C(α)_Val339_–C(α)_Thr203_, which varies from 28.6° (apo form) to 30.5° (Nb_10_–G-actin) and 35.6° (V_10_–G-actin) (Figure 3a). These evidences highly match with what was reported in the literature.^13^ The H-bond interactions observed by docking are almost retained along the whole simulation time. The differences are as follows: for V_10_ the interaction with Thr203 is replaced by Arg184, while for Nb_10_ that with Tyr69 is lost and the moiety slightly moves outside the cleft toward the subdomain II where Nb_10_ is rotated ∼90° compared to V_10_ on its principal axes (Figure 3b,c).

**Figure 4:**
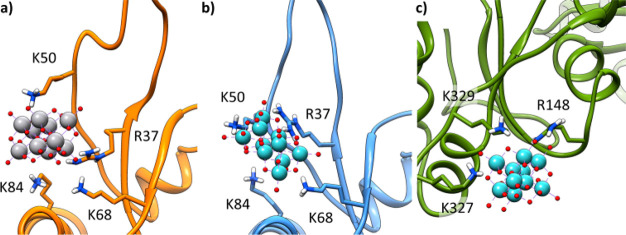
Molecular dynamics (MD) most sampled structures showing (a) the binding mode of V_10_ at the site β, (b) the binding mode of Nb_10_ at the site β, and (c) the binding mode of Nb_10_ at the site γ. The interacting side chains are explicitly depicted.

At the site β, both POMs form stable adducts during 200 ns of simulation. The H-bond interactions found by docking are generally retained with the addition in several frames of Lys50 coming from the flexible 39–51 loop (Figure 4a,b). The general G-actin folding is also maintained. The site γ shows differences between the two metal moieties: while the V_10_ leaves the surface of subdomain III at 75 ns and after 170 ns moves to the site β where it is stabilized, Nb_10_ forms a stable adduct during the whole trajectory. The H-bond network highlighted by docking is retained with the replacement of Thr148 with the positive side chain of Lys327 (Figure 4c). Finally, the site δ at the C-terminus is unstable for both the POMs. V_10_ at 40 ns moves away, and after 123 ns, it forms again a stable adduct at the site α. Nb_10_, due to its higher volume, shows a faster unbinding process and, in the first steps of the simulation, leaves the C-terminus to accommodate at the site γ, where it is stable along the entire trajectory.

From the energetic point of view the stable adducts formed by the two POMs at the binding regions of G-actin behave differently. While V_10_ shows the best affinity for the site α (Δ*G*_bind_ for the site α is lower than ca. 23 kcal mol^–1^ than the site β), the affinity of Nb_10_ for the sites α and β is comparable (according to our data, the site β is more stable than the site α of 2.0 kcal mol^–1^ and than the site **γ** of 17.2 kcal mol^–1^, Table 1). These results are in good agreement with the previous studies which suggested that V_10_ binds to proteins whose natural ligands are nucleotides, such as ribonucleases and myosin.^54^

**Table 1:** MD Residence Time of V_10_ and Nb_10_ at the Identified Sites (α, β, γ, and δ) and Binding Effects on the G-Actin Folding

site	subdomain(s)	V_10_ (*t*, ns)	Δ(Δ*G*_bind_)^a^	Nb_10_ (*t*, ns)	Δ(Δ*G*_bind_)^a^	fold
α	I/IV	stable, 200	0.0	stable, 200	2.0	φ variation^b^
β	II	stable, 200	22.9	stable, 200	0.0	retained
γ	III	unstable, <75		stable, 200	17.2	retained
δ	I/III	unstable, <40		unstable, <5		

a MM-GBSA relative values including quasi-harmonic entropic correction computed on 100 homogeneously distributed frames of the whole MD trajectories (values in kcal mol^–1^).

b φ = 28.6° (apo form), φ = 30.5° (adduct with Nb_10_), and φ = 35.6° (adduct with V_10_). The angle *φ* is defined in the text.

These results can rationalize the kinetic measurements of V_10_ decomposition as a function of G-actin concentration, monitored by UV/vis spectroscopy.^11^ In fact, G-actin causes marked effects in stability of V_10_, increasing its half-life from 5 h in solution up to 27 h (Figure S4); this demonstrates a clear protection effect exerted by G-actin toward V_10_. The best binding site proposed by simulations, the nucleotide site α, shows characteristics compatible with a protective environment being buried into the catalytic cleft of the proteins, and able to form a H-bond network, beside electrostatic interactions. Such interactions are strong enough to stabilize V_10_ into the pocket, preventing its exposition to the solvent and putative decomposition, as previously suggested.^11^

### Reduction of V_10_ to V^IV^ and Binding Sites of V^IV^O^2+^ Ion

**Figure 5:**
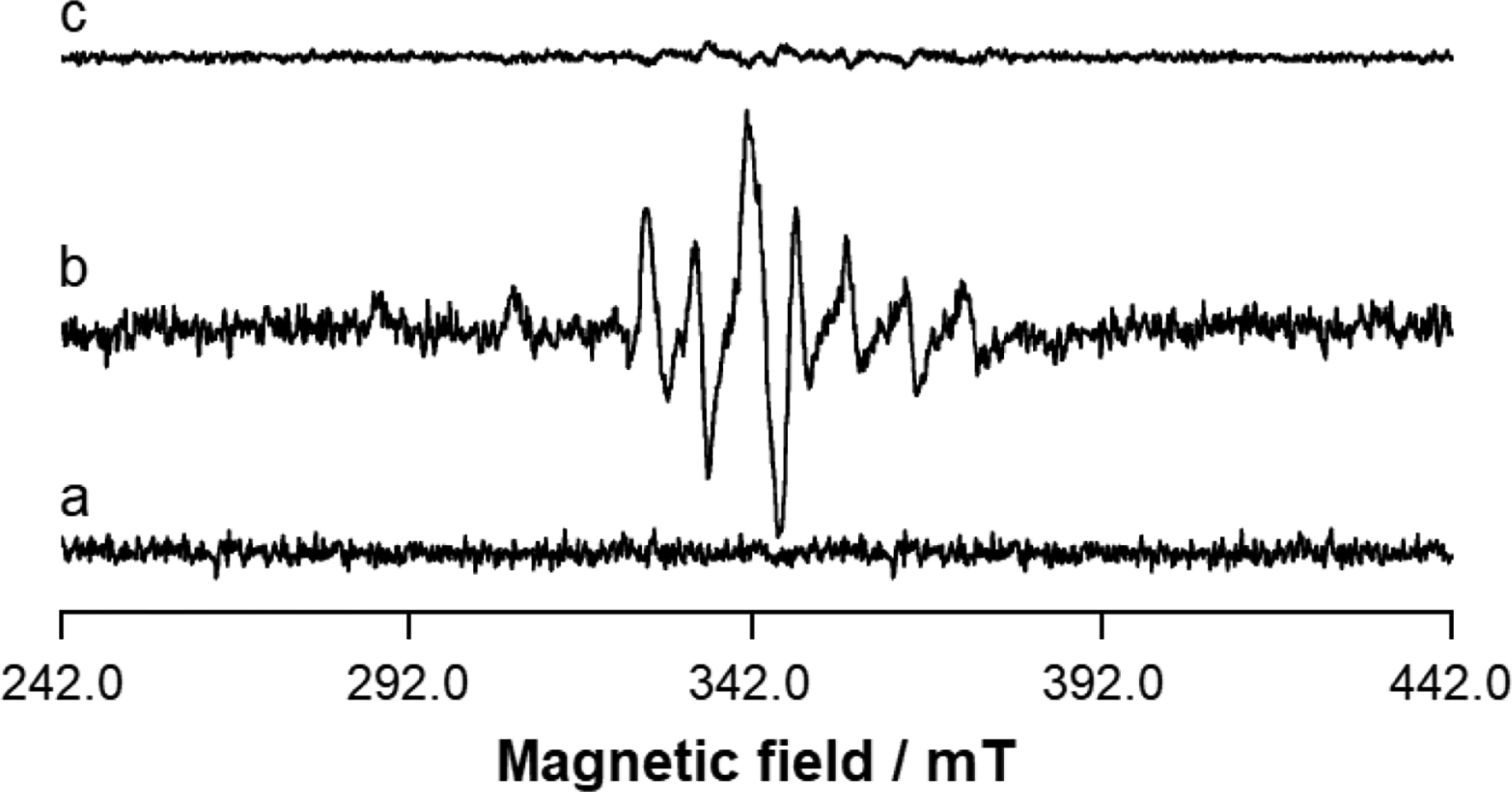
X-band EPR spectra recorded on frozen aqueous solution containing (a) 2.5 mM V_10_ in 2 mM Tris, 0.2 mM CaCl_2_, 0.2 mM ATP (pH 7.5); (b) 2.5 mM V_10_ in 2 mM Tris, 0.2 mM CaCl_2_, plus 100 μM G-actin (pH 7.5); and (c) 2.5 mM V_10_ in 2 mM Tris, 0.2 mM CaCl_2_, plus 50 μM G-actin and 0.2 mM of ATP to prevent oxidovanadium(IV) formation (pH 7.5). Adapted from refs (13 and 14).

After the incubation of V_10_ with G-actin, a rigid-limit EPR spectrum is revealed with eight resonances in the parallel region and eighth in the perpendicular one (trace b of Figure 5).^13,14^ This indicates a reduction process of V^V^ (3d^0^, EPR-silent) to V^IV^O (3d^1^, EPR-active), probably through the reaction of decavanadate(V) with the accessible −SH groups of cysteine residues. The most plausible candidates involved in the redox reaction are Cys272 and Cys374.^55^ The spin-Hamiltonian parameters for the adduct formed upon the binding of V^IV^O^2+^ to G-actin are *g*_*z*_ = 1.940 and |*A*_*z*_| = 177.1 × 10^–4^ cm^–1^;^14^ these values would indicate weak donors or—in agreement with the “additivity relationship”, the empirical rule verified for a lot of V^IV^O complexes which allows to determine the ^51^V hyperfine coupling constant along the *z*-axis, |*A*_*z*_|^estmtd^, from the sum of the equatorial donor contributions within ca. 3 × 10^–4^ cm^–1^ from the experimental value^56,57^—coordination of His residues with the aromatic plane perpendicular to the V^IV^=O bond.^56^ At the moment, the binding site and the residues interacting with V^IV^O^2+^ ion are not known; however, the effect of the addition of an excess of ATP (Figure 5c) suggests that the site could be the same as that of the nucleotide.

In this study, computational methods have been applied to determine the latent V^IV^O^2+^ binding site into the nucleotide binding region. The catalytic pocket was first probed for zones displaying at least two donor-containing residues with a sufficient backbone preorganization to bind V^IV^O^2+^.^29a,48^ A docking assay was performed, and two couples of amino acids emerged as potential donors, (His73; His161) and (His161; Asp154), both of them displaying the ability to coordinate V^IV^O^2+^ in a square pyramidal arrangement (Table 2).

**Table 2:** Full DFT Refined Structures (from the Docking Proposals), Estimated (estmtd) and DFT Calculated (calcd) |*A*_*z*_| Values, and Electronic Binding Energies for the Binding of V^IV^O^2+^ Ion to the Nucleotide Site α of G-Actin

donors	distances^a^	*F*_max_^b^	*F*_mean_^c^	pop. (%)^d^	Δ*E*_bind_^e^	|*A*_*z*_|^estmtd^ f	|*A*_*z*_|^calcd^ g
(N_H161_, COO^–^_D154_); O_W_; O_W_^h^	2.100, 1.964^i^	34.1	31.8	60	–28.4	178.4^j^	178.6^j^
(N_H73_, N_H161_); O_W_; O_W_^k^	2.128, 2.101^l^	45.4	39.5	64	–13.2	173.4^j^	169.4^j^

a Distance in Å.

b GoldScore *Fitness* value obtained for the more stable pose of each cluster.

c Average value of GoldScore *Fitness* for each cluster.

d Percentage computed considering the total solutions reported (numbers of solutions per cluster).

e Δ*E*_bind_ in kcal mol^–1^.

f |*A*_*z*_| estimated (estmtd) with the “additivity relationship” in 10^–4^ cm^–1^ units.

g |*A*_*z*_| calculated (calcd) with DFT methods in 10^–4^ cm^–1^ units.

h H_2_O molecules *cis* to each other.

i V–N_H161_ and V–O_D154_ distances, respectively.

j The experimental value of |*A*_*z*_| is 177.1 × 10^–4^ cm^–1^.

k H_2_O molecules *trans* to each other.

l V–N_H73_ and V–N_H161_ distances, respectively.

**Figure 6:**
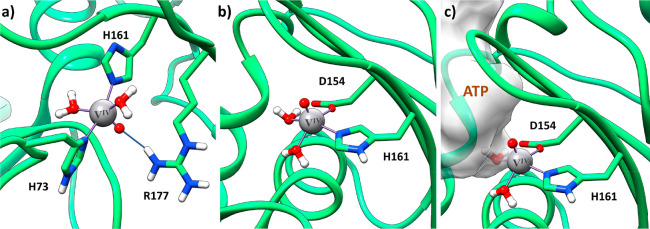
Full DFT optimized structures of the two docking proposals for the binding of V^IV^O^2+^ moiety to the nucleotide binding site α of G-actin: (a) the binding mode with (His73, His161), (b) the binding mode with (His161, Asp154), and (c) surface volume of ATP in its native site superimposed with the favored V^IV^O^2+^ binding. H-bonds are explicitly indicated with the blue lines.

The binding mode with two His [(His73, His161); O_W_; O_W_] is characterized by the *trans* arrangement of the two H_2_O molecules and by an additional H-bond stabilization between the oxido ligand and Arg177 (Figure 6a). The mode, in which His161 and Asp154 are bound to V^IV^O^2+^, displays a *cis* arrangement of the two aqua ligands with the aromatic ring of the His perpendicular to the V=O (θ, the calculated dihedral angle O=V^IV^–N–C, where C is the carbon atom bridging the two nitrogens, is 72.1°; Figure 6b). It results located deeper inside the nucleotide binding site α and overlaps significantly with the region with ATP (Figure 6c), making it a potential candidate to rationalize the V_10_/ATP competition observed by EPR. In fact, as reported by Ramos et al.,^13,14^ and described in Figure 5, the addition of ATP to a solution containing V_10_ and G-actin prevents the stabilization of paramagnetic V^IV^O^2+^, the product of the reduction of V_10_.

The predicted structures were refined at the full DFT level following the cluster method proposed by Siegbahn and Himo,^28^ and the QM binding energy was computed; finally, EPR spin-Hamiltonian parameters were calculated for the predicted sites. From an energetic point of view, both sites show negative Δ*E*_binding_ values, but that with (His161, Asp154) is favored by more than 15 kcal mol^–1^. Its |*A*_*z*_|^calcd^ (178.6 × 10^–4^ cm^–1^) matches well the experimental value of 177.1 × 10^–4^ cm^–1^ measured by Ramos et al.^14^ Moreover, |*A*_*z*_|^estmtd^, estimated with the “additivity relationship”,^56,57^ is 178.4 × 10^–4^ cm^–1^, closer to the experimental one than that expected for (His 73, His161) mode; the contributions of 45.1 × 10^–4^ cm^–1^ for His-N (derived from the equation 42.72 + 2.96 × sin[2θ – 90],^56^ θ being the calculated dihedral angle O=V^IV^–N–C, where C is the carbon atom bridging the two nitrogens), 42.1 × 10^–4^ cm^–1^ for Asp-COO^–^, and 45.6 × 10^–4^ cm^–1^ for water-O were used to determine |*A*_*z*_|^estmtd^.^56−58^

These findings fortify the hypothesis that the binding mode (His161, Asp73), in the internal region of the site α, is the best candidate to explain the experimental EPR data collected so far. In fact, the simultaneous binding of V^IV^O^2+^ and Ca^2+^-ATP results hindered for the steric clashes and electrostatic repulsion into the enzymatic cleft (Figure 6c).

### Competition between V_10_ and ATP or Nb_10_ for the Binding Sites

Some years ago Aureliano et al. showed that, while G-actin increases the stability of V_10_ in solution, the addition of ATP to the medium has the opposite effect, decreasing its half-life from 27 to 10 h (Figure S4).^11^ This insight suggested, for the first time, that V_10_ and ATP could compete for the same binding site, ATP being more affine to its natural site and able to partially displace V_10_ to a less protective and exposed binding region.

The possible competition between V_10_ with ATP for the nucleotide site was further evidenced in 2009 by EPR and ^51^V NMR spectroscopy.^13^ The EPR spectra recorded on frozen aqueous solution containing increasing ATP/V_10_ ratios in the presence and absence of G-actin indicated that V_10_ induces actin cysteine oxidation and formation of the paramagnetic oxidovanadium(IV) ion, and both the processes are prevented by the addition of ATP in solution (trace c of Figure 5). ^51^V NMR spectra recorded at room temperature on the same systems confirmed this finding and showed that it is necessary to increase the ATP concentration up to 5 mM to broaden the V_10_ signal.^13^ Moreover, the major alterations in protein structure suggested by ^1^H NMR spectra of G-actin treated with V_10_ (Figure S3), which are not or hardly visible in the system with ATP, confirmed the competition between nucleotide and decavanadate in the critical binding site.^50^

CD spectra show that G-actin is less stable in the absence (melting point, *T*_m_, of 44.8 °C) than in the presence of ATP (*T*_m1_ 48.9 and *T*_m2_ 64.3 °C, *vide supra*), whose interaction results in two melting points which were extracted from recorded melting curves. Finally, recent EXAFS and XANES experiments established that the interaction of V_10_ with G-actin triggers a protein conformational reorientation that causes an oxidation of Cys residues and formation of V^IV^O^2+^. The variations induced in V_10_’s EXAFS profile in the presence of ATP suggest that the nucleotide and V_10_ compete for the same binding site (see the EXAFS profile in Figure 2).^15^

To deduce whether the native actin substrate (ATP) interferes with V_10_ binding and to rationalize the experimental evidence (*vide supra*), additional MD simulations were carried out. ATP is found at the enzymatic cleft of G-actin in correspondence of the site α and, in principle, could compete with V_10_ for this region. From a first MD of 200 ns, the binding of Ca^2+^-ATP to the site α is highly stable along the whole trajectory. The Δ*G*_bind_ of Ca^2+^-ATP to its biological site α is the lowest of the series and is more favorable by 14.1 kcal mol^–1^ than V_10_.

It must be highlighted that the catalytic cleft is more closed than the apo or POMs loaded forms with an angle φ of 22.0° (cf. data in Table 1). This observation is coherent with ^1^H NMR spectra of G-actin recorded in the presence and absence of V_10_. Major alteration patterns attributable to a protein conformational change are negligible upon addition of ATP (Figure S3).^52^ Therefore, ATP could displace V_10_ from the site α, causing the closure of the cleft to its natural conformation and giving the normal pattern observed in ^1^H NMR experiments.

**Figure 7:**
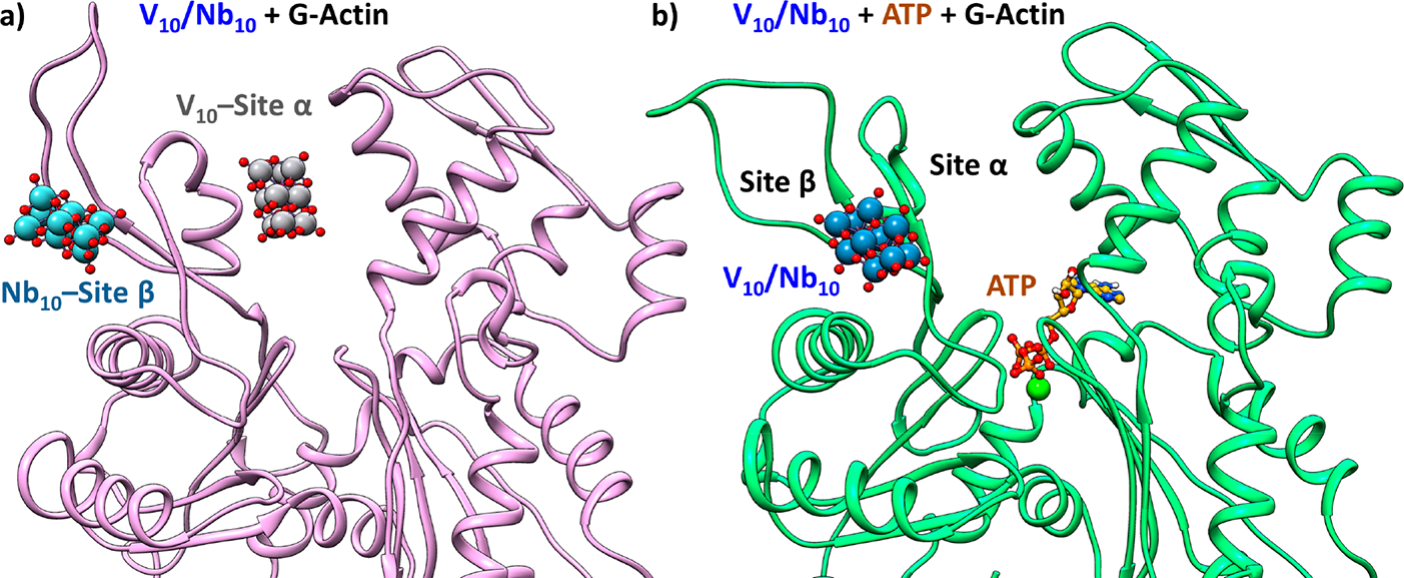
Molecular representation of (a) the contemporaneous binding of V_10_ at the site α and Nb_10_ at site β and (b) the binding mode of V_10_ and Nb_10_ at the site β in the presence of Ca^2+^-ATP.

Aimed to prove this hypothesis, further MDs were performed. On the most sampled structure of the trajectory with Ca^2+^-ATP bound to the site α, a subsequent docking of V_10_ was performed to evaluate whether the contemporaneous binding of V_10_ and ATP at the site α is possible. An additional 200 ns of MD unveils that the presence of ATP hinders the V_10_ binding; indeed, at 9 ns, V_10_ leaves the site α and reaches the site β after 53 ns, where it is stabilized (Figure 7).

These data can be rationalized assuming that in the case of V_10_ the effect of ATP is of clear inhibition. The catalytic site becomes populated preferentially by ATP with concomitant closure of the cleft which, even for the increased negative charge in the region, induces the displacement of V_10_ from the site α to the site β less affine of ca. 23 kcal mol^–1^ (see Table 1).

As discussed above, the site β is found at the interface of subdomains I and II in the surface region of G-actin (Figure 1a). Therefore, the reason behind the increasing of the half-life of V_10_ bound to G-actin from 5 to 27 h and its decreasing up to 10 h upon addition of ATP^11^ should be found on the ATP-induced displacement of V_10_ from the protective environment of the site α to the more solvent exposed site β. This event may favor the Cys oxidation and V_10_ reduction to oxidovanadium(IV) cation, V^IV^O^2+^, and the aggregation of G-actin to form F-actin observed in recent EXAFS and XANES study.^15^

Concerning Nb_10_, being its affinity for the sites β and α comparable, the competition with ATP is less important; in fact, Nb_10_ can be hosted at preferred site β in the presence of the nucleotide. Based on these consideration, no competition between the two POMs and G-actin is expected (Figure 7). This finding allows to establish a different behavior with respect to what was observed for the binding with Ca^2+^-ATPase; in that system, Nb_10_ prevented the binding of V_10_, indicating that both isostructural POMs share the same primary binding site in the protein structure.^18,19^

## Conclusions

This study represents an unusual attempt to rationalize the experimental data—collected over the past 15 years—between the interaction of a POM, decavanadate(V) or V_10_, and a very important cellular protein, G-actin, through computational methods. In fact, despite various instrumental techniques being able to ascertain the binding between a potential metallodrug and a protein, nevertheless they often cannot provide the description of this interaction at the molecular level. For the system V_10_–G-actin, the binding sites and interacting residues are not known nor is the reason for the competition with other molecules of the organism, for example, the physiological ligand ATP. In this context, molecular modeling represents a valid tool to understand these processes, providing new information for the comprehension of the molecular events governing POMs–proteins and, in general, metallodrugs–protein binding.

For a careful application of POMs in biology and medicine there is a great need for understanding which cellular proteins represent the potential targets, and often POMs–proteins interaction did not get the attention it deserves. Specifically, regarding POMs–actin systems described in this study, the computational approach has proven to be an excellent tool to determine the binding sites and understand the different V_10_ and Nb_10_ modes of action. Summarizing the obtained results, it was elucidated and compared at the molecular level the behavior of Nb_10_ and V_10_ in the G-actin conformational rearrangements. Two putative V_10_/Nb_10_–actin binding sites were fully characterized: V_10_ prefers the catalytic nucleotide site (site α), whereas Nb_10_ is more stable at the site β. Therefore, in contrast with other proteins such as Ca^2+^-ATPase, they could contemporaneously bind to G-actin, and their biological and/or pharmacological action could be synergistic. These findings should be considered for other studies in inorganic medicinal chemistry, administering POMs or other potential metallodrugs compatible with the same target protein, that is, with affinity for different sites, to amplify their action. Concerning the presence of the native actin ligand ATP, it prevents the V_10_ binding to the site α, whereas it does not affect Nb_10_–actin interaction, since β is the primary site for decaniobate.

We believe that the present paper will facilitate a deeper understanding of the role POMs in various applications, giving valuable insight for a rational design of new metal-substituted POMs with specific interaction properties toward the putative target proteins. In addition, it could be used as a “guide” for the study of the interactions (metal compounds)–(cellular proteins) and of the phenomena of inhibition and competition with the biomolecules at physiological conditions.

Finally, an important outcome of this paper is that other metallodrug–protein systems, in the absence or presence of eventual inhibitors and/or competition with molecules of the organism, could be studied with the same approach, suggesting important elements to explain the biological data and design more active and specific potential drugs.

## Supporting Information Available

Comparison of the DFT optimized structure of V_10_ and Nb_10_ (Figure S1), exchange rate and apparent dissociation constant for the exchange of bound ε-ATP of G-actin with ATP (Figure S2), ^1^H NMR spectra of G-actin and V_10_–G-actin (Figure S3), V_10_ half-life time in the presence of G-actin and ATP (Figure S4), and Cartesian coordinates of V_10_ and Nb_10_ (PDF)
