# Supplementary material for: Rationalizing the Decavanadate(V) and Oxidovanadium(IV) Binding to G-Actin and the Competition with Decaniobate(V) and ATP
Source: Inorg Chem. 2020 Nov 30;60(1):334–44. doi: 10.1021/acs.inorgchem.0c02971 (PMC8016201; doi:10.1021/acs.inorgchem.0c02971)
Supplement: Supplementary file 1 — ic0c02971_si_001.pdf [file ic0c02971_si_001.pdf]

SUPPORTING INFORMATION

Rationalizing the Decavanadate(V) and Oxidovanadium(IV) Binding to G-actin and the Competition with Decaniobate(V) and ATP

Giuseppe Sciortino,[§,#] Manuel Aureliano,[†] and Eugenio Garribba,[§]*

[§] Dipartimento di Chimica e Farmacia, Università di Sassari, Via Vienna 2, I-07100 Sassari, Italy

[#] Institute of Chemical Research of Catalonia (ICIQ), 43007 Tarragona, Spain

[†] CCMar, FCT, Faculdade de Ciências e Tecnologia, Universidade do Algarve, 8000-139 Faro, Portugal

Corresponding authors. E-mail: gsciortino@iciq.es (G.S.); maalves@ualg.pt (M.A.); garribba@uniss.it (E.G.).

1. Supplementary figures

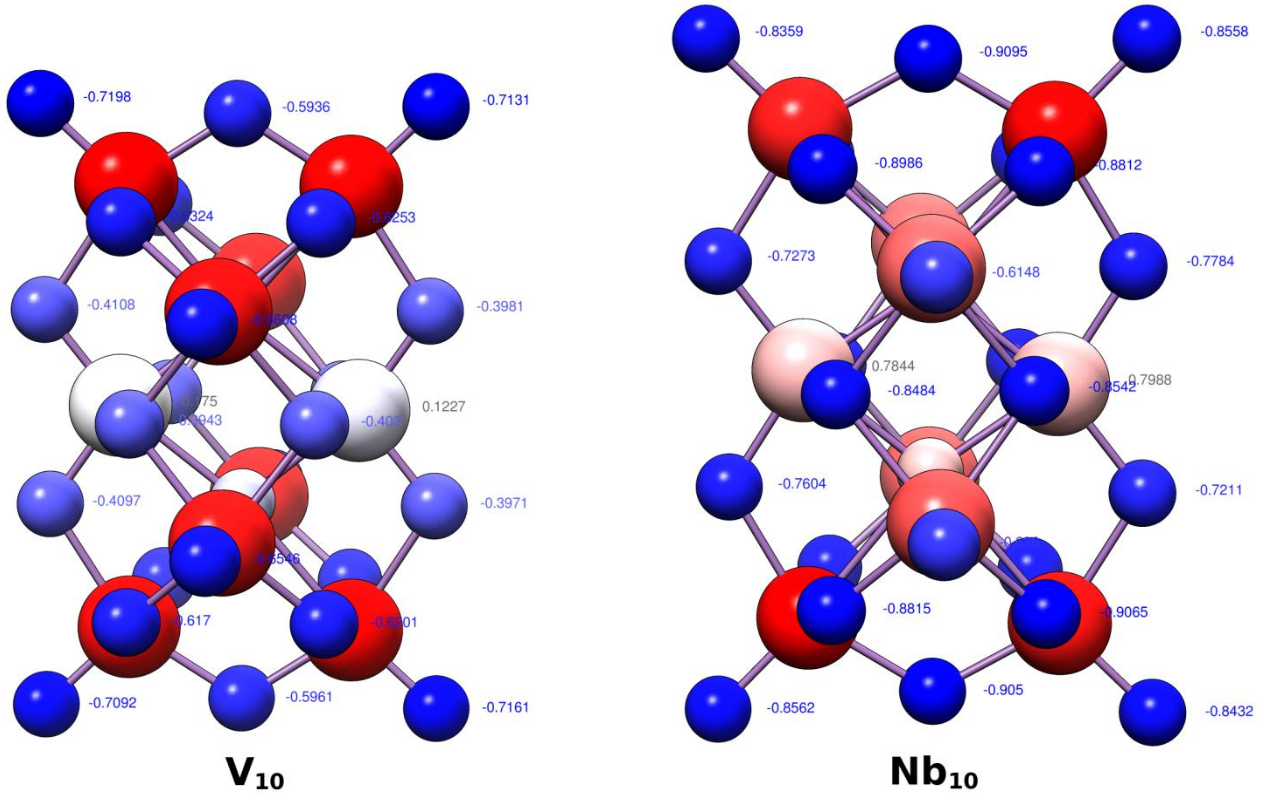

Figure S1. Comparison of the DFT optimized structure of V_{10} and Nb_{10}. Surface atomic RESP charges are shown. Color code: V is shown in red and white, Nb in red and light pink and O in blue.

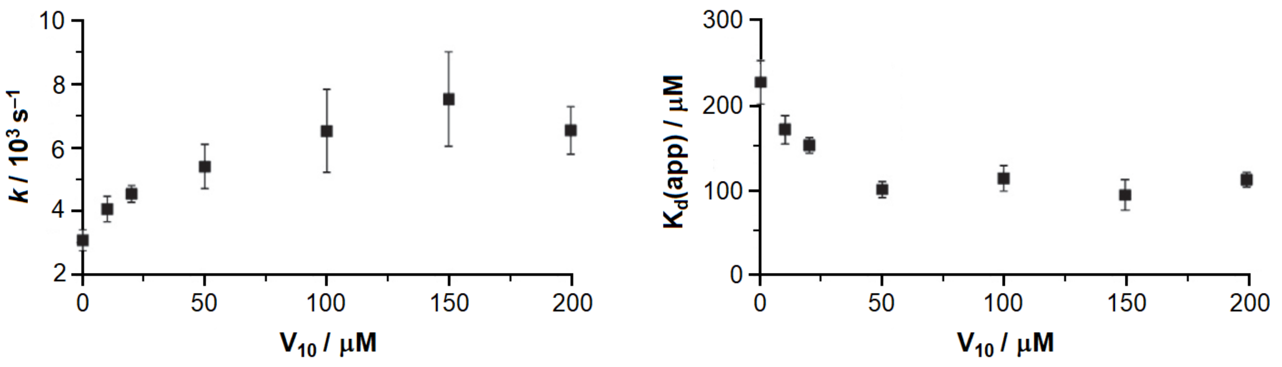

Figure S2. Exchange of bound ε-ATP of G-actin with ATP as a function of V_{10} concentration: a) the ε-ATP exchange rate *k*; b) the apparent dissociation constant K_d(app). Actin monomers (5 µM) were incubated for 20 min with 0-200 µM decavanadate in 2 mM Tris (pH 7.5), 0.2 mM $CaCl_2$. The calculated value of *k* was 6.5×10^{-3} s^{-1} and were of K_d(app) of 227.4 ± 25.7 µM and 112.3 ± 8.7 µM in absence or presence of 200 µM decavanadate. Adapted from ref. 1.

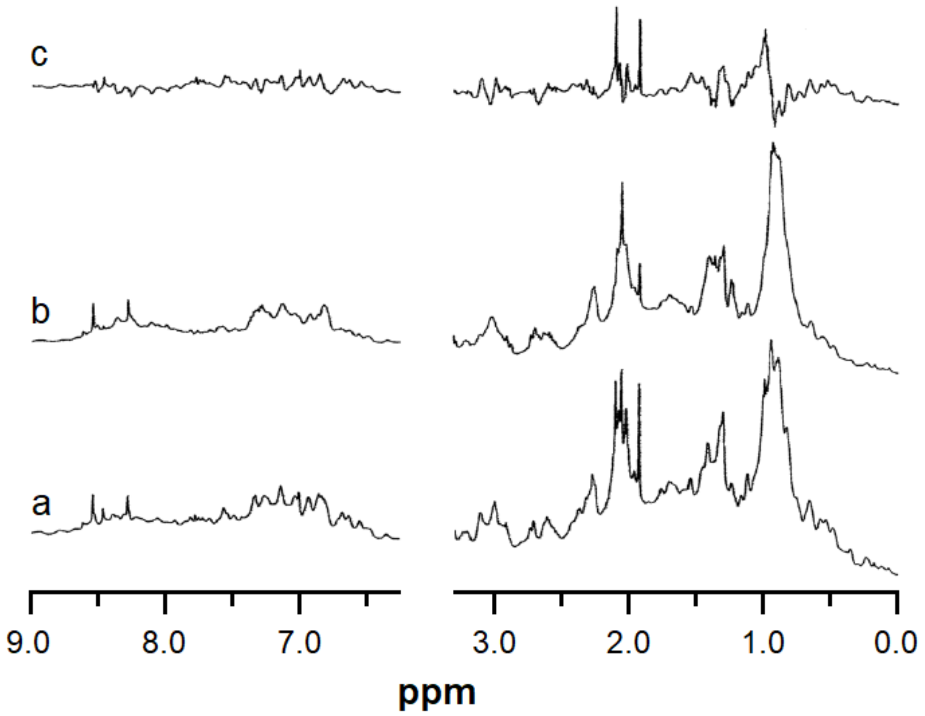

Figure S3. ^{1}H NMR spectra of: a) 63 μM G-actin and b) 63 μM G-actin plus 0.1 mM V_{10}. In the trace c the difference spectrum are shown. The spectra were obtained in the medium containing 2 mM Tris–HCl (pH 7.5), 0.2 mM $CaCl_2$. The addition of 0.2 mM of ATP to G-actin results in a negligible change of the spectrum. Adapted from refs. 2 and 3.

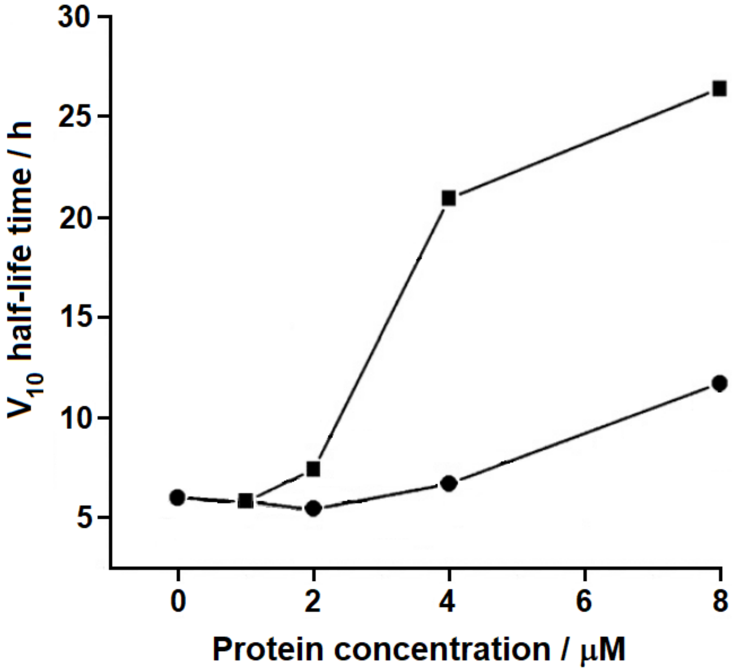

Figure S4. Decavanadate half-life time in presence of G-actin: without ATP (black squares) the data on the system containing 2 mM Tris, 0.2 mM $CaCl_2$, 10 μM ATP, 100 mM KCl, 2 mM $MgCl_2$, pH 7.5, and with ATP (black circles) the data on the system containing 2 mM Tris, 0.2 mM $CaCl_2$, 0.2 mM ATP, 100 mM KCl, 2 mM $MgCl_2$, pH 7.5. Adapted from ref. 4.

2. Cartesian coordinates

5.1. Decavanadate ($V_{10}O_{28}^{6-}$; V_{10})

V	-3.096682000	-0.018323000	-1.520436000
V	-1.539839000	2.230713000	-0.026008000
V	-0.036383000	-0.021241000	-1.643581000
V	-1.545296000	-2.228969000	0.015013000
V	-3.053863000	0.021746000	1.567589000
V	3.096669000	0.018423000	1.520543000
V	1.539852000	-2.230828000	0.026089000
V	0.036377000	0.021258000	1.643551000
V	1.545287000	2.228965000	-0.015066000
V	3.053866000	-0.021805000	-1.567572000
O	-4.196022000	-0.015961000	-2.688006000
O	-2.699948000	1.811105000	-1.331156000
O	-1.360566000	-0.000195000	-2.679747000
O	-2.701503000	-1.816540000	-1.325533000
O	-4.055524000	0.000669000	0.018463000
O	-1.304368000	-0.000759000	-0.022499000
O	-0.004062000	-1.868669000	-1.240463000
O	-0.005244000	1.865846000	-1.234449000
O	-2.684150000	1.812442000	1.330740000
O	-2.684731000	-1.806395000	1.337303000
O	-1.530101000	3.839510000	-0.016927000
O	1.341585000	0.002150000	-2.642924000
O	-1.533420000	-3.837973000	0.012538000
O	-4.146649000	0.019501000	2.738579000
O	4.195963000	0.015675000	2.688132000
O	2.700057000	-1.811167000	1.330752000
O	1.360585000	0.000258000	2.679736000
O	2.701532000	1.816519000	1.325493000
O	4.055509000	-0.000623000	-0.018358000
O	1.304327000	0.000972000	0.022462000
O	0.004024000	1.868698000	1.240440000
O	0.005254000	-1.865842000	1.234440000
O	2.684233000	-1.812430000	-1.330782000
O	2.684698000	1.806412000	-1.337314000
O	1.529996000	-3.839677000	0.016310000
O	-1.341574000	-0.002235000	2.642907000
O	1.533443000	3.837966000	-0.011955000
O	4.146690000	-0.019077000	-2.738534000

5.2. Decaniobate ($Nb_{10}O_{28}^{6-}$; Nb_{10})

Nb	3.453586000	0.000113000	-1.636229000
Nb	1.671398000	-2.418486000	0.002806000
Nb	0.163285000	0.000039000	-1.724624000
Nb	1.671049000	2.418169000	0.002918000
Nb	3.250408000	0.000411000	1.802587000
Nb	-3.453553000	-0.000105000	1.636218000
Nb	-1.671385000	2.418528000	-0.002762000
Nb	-0.163310000	-0.000026000	1.724550000
Nb	-1.671037000	-2.418235000	-0.002860000
Nb	-3.250371000	-0.000391000	-1.802567000
O	4.737096000	0.000861000	-2.860082000
O	3.000131000	-1.951187000	-1.353836000
O	1.704817000	0.000236000	-2.822276000
O	2.999648000	1.951643000	-1.353667000
O	4.333217000	0.000089000	0.134798000
O	1.287877000	0.000412000	0.052939000
O	0.027567000	1.989853000	-1.301864000
O	0.028335000	-1.989902000	-1.301983000
O	2.849414000	-1.939753000	1.477815000
O	2.850434000	1.940591000	1.477375000
O	1.697596000	-4.194885000	0.018564000
O	-1.398973000	-0.000906000	-2.805226000
O	1.694694000	4.194660000	0.017569000
O	4.472373000	0.000121000	3.090628000
O	-4.737077000	-0.000861000	2.860064000
O	-3.000185000	1.951248000	1.353794000
O	-1.704863000	-0.000203000	2.822258000
O	-2.999642000	-1.951699000	1.353696000
O	-4.333158000	-0.000083000	-0.134750000
O	-1.287852000	-0.000385000	-0.052959000
O	-0.027573000	-1.989856000	1.301901000
O	-0.028373000	1.989911000	1.302024000
O	-2.849430000	1.939732000	-1.477799000
O	-2.850327000	-1.940563000	-1.477365000
O	-1.697661000	4.194913000	-0.018708000
O	1.398897000	0.000902000	2.805157000
O	-1.694916000	-4.194722000	-0.017693000
O	-4.472420000	-0.000262000	-3.090563000

3. References

(1) Ramos, S.; Moura, J. J. G.; Aureliano, M. Actin as a potential target for decavanadate. *J. Inorg. Biochem.* **2010**, *104*, 1234-1239.

(2) Ramos, S.; Almeida, R. M.; Moura, J. J. G.; Aureliano, M. Implications of oxidovanadium(IV) binding to actin. *J. Inorg. Biochem.* **2011**, *105*, 777-783.

(3) Ramos, S.; Moura, J. J. G.; Aureliano, M. Recent advances into vanadyl, vanadate and decavanadate interactions with actin. *Metallomics* **2012**, *4*, 16-22.

(4) Ramos, S.; Manuel, M.; Tiago, T.; Duarte, R.; Martins, J.; Gutiérrez-Merino, C.; Moura, J. J. G.; Aureliano, M. Decavanadate interactions with actin: Inhibition of G-actin polymerization and stabilization of decameric vanadate. *J. Inorg. Biochem.* **2006**, *100*, 1734-1743.
